# Supplementary material for: Modeling opinion misperception and the emergence of silence in online social system
Source: PLoS One. 2024 Jan 11;19(1):e0296075. doi: 10.1371/journal.pone.0296075 (PMC10783706; doi:10.1371/journal.pone.0296075)
Supplement: Attachment — Submitted filename: VP2023_Responses2reviewers.pdf [file pone.0296075.s001.pdf]

Responses to Reviewers' Questions

Comments to the Author

Reviewer #1:

This study describes (computer) simulation experiments to explore different theoretical pathways that can explain the empirical finding of "misperception" – that is, humans' erroneous impression of how a certain opinion or belief is distributed in the population. I list here my comments – I put them together by theme, generally trying to list the more general ones at the top and leaving the more secondary ones at the bottom.

On the substantive level, I invite the authors to reflect on the limitations of their study, particularly on how dependent their results are on key modeling assumptions. I will make three examples. My recommendation is to surface and motivate these assumptions upfront in the manuscript (e.g. where the model is introduced) or at least to surface and comment on them in a wider discussion on the limitations and generalizability of the study at the end of the manuscript.

We thank the reviewer for the suggestions made. See below for a point-to-point response.

- One of the unappreciated key assumptions, I believe, is that privately held beliefs (denoted b) are fixed. This implies that voiced opinions can change, whereas private opinions do not (or, if they do, they can change at a much slower rate such that it is not captured by the model). As far as I know this assumption is quite unusual in the literature and needs to be motivated. It seems reasonable to expect that results would be different if individuals adjusted their private beliefs, too: I say this with an eye on literature on behavioral mechanisms such as conformity and cognitive dissonance, where human tendency to conform with the perceived norm is not only superficial (read: it does not only affect publicly voiced opinions), but it may actually affect one's privately held belief, too. If so, I would expect misperception to be a less prominent emerging feature of the model.

We thank the reviewer for the comment. The body of literature modeling spiral of silence and related mechanisms (e.g, normative social influence and pluralistic ignorance) typically assumes beliefs as fixed (e.g., Ma & Zhang 2021, Ross et al 2019, Cabrera et al 2021, Sohn & Geidner 2016, Wu et al 2015, Merdes 2017). As in our case, such a choice is justified by the fact that the primary focus of these models (and the theories behind) is not about people changing their private beliefs, but rather the process that leads individuals to express opinions that do not align with their privately held ones. In this sense, having changing beliefs is not a key requirement. Besides helping to isolate the specific mechanisms related to opinion misperception, one may also argue that having fixed private beliefs can reflect a more realistic representation of individuals' long-term values, attitudes, or opinions. Indeed, as also suggested by the reviewer, in many real-world scenarios people's core beliefs tend to

remain relatively stable over time. We anyhow acknowledge that such a modeling choice is a simplification that may not be able to capture all aspects of real-world opinion formation and change. We explicitly added this point as a limitation, see lines 455-460.

- Another key assumption is about the social influence mechanism implemented in the model. Page 4 line 122 introduces what is essentially a "majority rule" model where the object of influence is not agents' privately-held opinion but the opinion they opt to voice. Different social influence mechanisms are likely to produce different macro-level outcomes. Readers should be made aware that these results only apply to the extent that individuals' choice as to which opinion to express follows a majority rule mechanism. (Incidentally, the authors are also advised to cite the relevant literature on the majority rule model – currently lacking).

We thank the reviewer for raising this point. The mechanism implemented, which indeed can be seen as a modification of the majority rule, was chosen in accordance with the relevant social psychological theories explaining misperception phenomena. Specifically, it rests on the assumption that agents care about (and thus may conform to) what they perceive "most others" think, regardless of whether such a perception is correct. Since such a perception can only be formed based on what they actually observe (i.e. neither the global distribution of expressed opinion nor that of private beliefs is known to agents), this mechanism becomes very suitable, both practically and theoretically, to explore how misperception emerges as a macro-scale outcome. Nevertheless, we agree that implementing different rules to characterize social influence would help clarify the generalizability of our findings. We have added specific citations of the majority rule in line XXX and included reviewer's point in line (see lines 142 and 460-463).

- There is another assumption that might deserve some consideration – but which might be less important for this study and more important for future work: the granularity/dimensionality of the opinion scale. Is it reasonable to expect results to change if the opinion scale had more levels than two? And if it was multidimensional (e.g. if individuals simultaneously had more beliefs about related things and could decide which one to voice when interrogated?)

In our case, the two-option model was chosen because it represents a good approximation of a simple scenario involving polarizing debates in social media, as for instance pro/anti vaccine cases. Nevertheless, we agree with the reviewer that the granularity of the opinion scale, the number of levels or categories it contains, could impact the outcomes and dynamics observed. For example, when opinions can be expressed at multiple levels individuals may find it easier to identify common ground or moderate positions, and stronger evidence or more substantial differences with respect to their social surroundings could be required to start a silencing process. When accounting for multidimensionality, individual's opinions on similar topics may not evolve independently because of the logical connections an individual places between them. Such an interdependency may also affect the speed with

which certain opinions stabilize within a community. We included these observations to stimulate future works in this direction (lines 470-481).

Another general comment I have – weighing negatively in my evaluation of the manuscript – concerns the form. The most problematic issue in this regard is that key concepts are lacking a proper definition and are referred to using different inconsistent labels. This makes the manuscript difficult to understand where it matters most. I make here some examples – note that the examples I chose are particularly delicate in that they involve some main concepts in the study whose meaning should be unambiguous.

- Behavior / decision / opinion / position / belief / information: these terms seem to be used interchangeably in the introduction, to the point that it is not clear if and when the use of one instead of the other is meaningful.

We thank the reviewer for this comment. In the introduction we try to bridge between different disciplines that tend to use different terms for potentially similar concepts. For example, work in social psychology and related fields may refer to the dichotomy of behaviour vs belief to denote a distinction between what is publicly observable (others' behaviour) and what is inferrable from it (others' belief). In this sense, it shares many analogies with the dichotomy of expressed vs private opinion in media communication studies (and in related ABM studies). We made considerable changes in the text aiming at providing more coherent references to the key concepts, which are written in red in the manuscript with track changes. For example, we avoided as much as possible what we feel are synonyms for opinions (e.g., position, views), and limited references to behaviour when possible. We also clarify that "beliefs" and "private opinions" are meant as overlapping concepts in our model definition (see a more elaborated response below). We hope that the description of the key concepts in present form of the manuscript has reached a satisfying level of coherence and consistency.

- Contrarian / minoritarian agent seem to mean the same thing. Furthermore, not only the label is inconsistent: the underlying definition of contrarian is problematic. On page 2, line 54 contrarians are defined as "individuals holding extreme positions". Opinions in this study are modeled as essentially binary. How can an opinion on a binary scale be "extreme"? In a binary opinion scale all agents will have an opinion at the extreme end of the scale, so they all will be "holding extreme positions". Note that on page 5, line 162 a new definition of "contrarians" is introduced: "committed users holding unpopular opinions". This works better, but it still isn't perfect: what does "committed" mean in this context, and is it really necessary for this definition? Furthermore, immediately following the new definition (line 165) from the implementation of contrarians in the model it becomes apparent that the essence of being a contrarian is not so much about the opinion being held, but the consistency with which contrarians voice their true opinion (opinion strength is set to 1 for contrarians).

We agree with the reviewers that the definition of contrarians may sound problematic. In our model the key elements of such agents are that a) they hold an unpopular opinion (in this sense, they represent a “minority”), and that b) publicly stick to their opinion regardless of the prevailing opinion climate. That is, they always express a public opinion which is in line with their privately held one. Given that point b) recalls the definition of “hard-cores” by Noelle and colleagues, we now refer to them as “hard-core minority” (we introduced the term at lines 83-87 and used throughout the text, written in red in the manuscript with track changes) and use this term consistently throughout the paper, deleting potentially confusing adjectives (e.g., committed, extreme).

- Misperception / social bias seem to mean the same thing. If not, the first occurrence of “social bias” (line 23) makes the meaning of this paragraph obscure.

It is correct. We now choose to refer only to misperception to improve consistency (first occurrence at line 26).

Incidentally, the sentence on lines 22-24 has other issues. It is also unclear what “online social system” refers to;

The sentence has been rephrased (line 25-28).

and the sentence tries to introduce too many ideas at once: (1) that there is empirical evidence of misperception; (2) that there is some theoretical puzzle concerning the empirically-observed misperception; (3) that misperception -- and/or the theoretical puzzle about it -- involves online social networks. If I understand correctly, this sentence can be rewritten with less ambiguity as follows: "Despite empirical evidence of misperceptions about the popularity of certain behaviors and opinions -- particularly in online social networks [refs], -- we still lack a clear understanding of how these misperceptions emerge in the first place. "

We revised the text according to the reviewer's suggestions (lines 25-28).

- Opinion expression / opinion declaration / and other various circumlocutory expressions also seem to mean the same thing.

We revised the text accordingly (modifications in red in the manuscript with track changes.

Other more secondary comments:
- Plos One submission guidelines mandate, with few exemptions, the publication of research data in public repositories. The data statement for the manuscript seems insufficient in this

regard, as replication scripts are made available upon request to the authors whereas they should be deposited to a public repository.

We now included the codes to generate data as supplementary material.

- About the first equation (page 10): square brackets are here used to signify some operation on the sum of w over all neighbors of i. I am clueless as to what that operation is (the mode perhaps?). Please check for correctness and consider adding explanatory text.

Indeed. There was a typo, a “sign” was lacking in the text, and the brackets simply refer to the sign function. We fixed the typo and added the definition of the function sign[.] in the text.

- Line 64: This is the first occurrence of the terms “exogenous” and “endogenous” to refer respectively to illusion of majority and pluralistic ignorance. However, I do not get why one would be exogenous and the other endogenous. I can understand they are different phenomena -- one is a feature of social networks and the other is a social phenomenon -- but I don't clearly see how endo- or exogeneity captures the essence of their difference.

We thank the reviewer for his/her observation. Throughout the paper, we use the term “exogenous” consistently to refer to those sources of misperception that do not depend on the agents’ behavior, but merely on their dislocation in the network (e.g., friendship paradox), while we use “endogenous” to refer to a source of misperception that is mostly driven by agent’s behavior, more specifically by how the agent incorporates the social information in this decision-making process (e.g., the evaluation of the cognitive cost of declaring its belief according to the observable social context). Following this distinction, referring to exogenous sources is useful to highlight the contribution to misperception stemming from a discrepancy between local and global landscape, while endogenous ones can highlight the one stemming from a public-private discrepancy. We have clarified this point in the Introduction, (lines 66, 71,73-74).

- About Figures. Some figures are very busy / information dense. Information density is not a problem, but it requires holding the reader by hand. Labelling all axes is a must. High(er) pixel density/resolution will be necessary. Using colorblind friendly color palettes is recommended, particularly when the line style or pointer shape do not disambiguate between elements (avoid e.g. purple vs black in Figure 1 right; and red vs green in Figure 2). Making sure that figure captions are close to the actual pictures is a welcome help to reviewers.
About figure 1 specifically: is there a reason why misperception and public vs private mismatch are only highlighted for the black lines? If the only reason is that purple lines do not allow for enough space, perhaps the highlight in the plot can be replaced with an

explanation in-text or in caption. And on the right panel, the X-axis label reads “K_0”. Should it not be “K_c”?

We thank the reviewer for the remark. We improved the quality and clarity of the figures. We have changed them and made them all black and white, uniformed the legends and captions to make them cleaner and more coherent with the main text. Moreover, we have used eps format instead of png to increase the resolution.

- Line 30: “support the role of” -> perhaps you mean “confirm the importance of”

We have revised the text accordingly (line 34).

- Line 35: what is meant by "understanding of the origin of the information"? Do you mean "understanding of the truthfulness of the information", "trustworthiness of the person who is sharing the information", "whether the information is truly held/believed by many and not just very minoritarian", or something else?

The sentence has been simplified and the referred text is no longer there.

- Line 37: “Observational data […]”. I can understand what you mean because I know that there is a literature on opinion dynamics and I agree that it can be used to model the emergence/spread of misperception and ultimately polarization. However, the generalist reader will be clueless at this point. You mention behavioral changes and complex dynamics, but the introduction at this point has only mentioned phenomena such as sharing/receiving information/opinions and estimating how widespread in society such information/opinion is. These phenomena per se do not involve any behavior change or complex dynamics. Therefore, it is not clear at this point what dynamics the text is referring to.

The sentence has been reformulated avoiding the use of such confounding expression (lines 41-46).

- Lines74-75: “[…] when a committed minority […]”. This sentence is unclear. Please check for syntax. What I understand: "when a community of agents -- agreeing, to varying degrees, on a moderate opinion -- is joined by a small group of contrarians."

We have revised the text accordingly (lines 78-80).

- Line 94. I do not understand what the word “heterogeneous” means here.

We deleted the term, as it is specified later in the text.

- Line 106: "[…] σ_i: this is the only variable describing the internal state of the agents". Is b_i -- the private opinion – not also an internal state of agents?

Correct. We deleted the misleading sentence.

- Line 111: "see below for clarifications". I have not seen or recognized what clarification this is referring to. The first concept that I think relates to w_i=0 is silence – which comes into play much later in the manuscript.

We clarified the meaning in the text (see lines 124-127).

- Line 176: "prestige". How prestige relates to degree requires a bit of explanation. An inexpensive solution perhaps is to say "centrality" instead?

We changed the term accordingly, and clarified its (metaphorical) meaning in lines 273-274.

****

<u>Reviewer #2:</u>

This paper addresses the investigation of opinion misperception in the context of social media, a phenomenon exacerbated by the silence of a small yet highly influential group of users.
Numerical simulations are shown to highlight the influence of the connections structure on the distortion of the perception of individuals' belief systems.
Given the substantial impact of this phenomenon on opinion dynamics, it is a subject worthy of investigation. The paper may be considered for publication after addressing the following points:

1- Pag.2: In the Introduction some crucial literature is missing. For example the concept of contrarian agent is introduced without taking into account the works done in this framework by Serge Galam and his collaborators. Moreover the authors say regarding the ABM models that "they are less suited to examine the processes by which a small fraction of individuals holding extreme positions (hereby "contrarians") can fuel opinion misperception...". In this framework, I would suggest to check and compare the following papers:
- Galam, Serge. "Contrarian deterministic effects on opinion dynamics:"the hung elections scenario"." Physica A: Statistical Mechanics and its Applications 333 (2004): 453-460.
- Borghesi, Christian, and Serge Galam. "Chaotic, staggered, and polarized dynamics in opinion forming: The contrarian effect." Physical Review E 73.6 (2006): 066118.
- Jacobs, Frans, and Serge Galam. "Two-opinions-dynamics generated by inflexibles and

non-contrarian and contrarian floaters." Advances in Complex Systems 22.04 (2019): 1950008.
- Galam, Serge. "From 2000 Bush–Gore to 2006 Italian elections: voting at fifty-fifty and the contrarian effect." Quality & quantity 41.4 (2007): 579-589.
- Javarone, Marco Alberto. "Social influences in opinion dynamics: the role of conformity." Physica A: Statistical Mechanics and its Applications 414 (2014): 19-30.
- Nyczka, Piotr, and Katarzyna Sznajd-Weron. "Anticonformity or independence?—Insights from statistical physics." Journal of Statistical Physics 151.1-2 (2013): 174-202.
- Iacomini, Elisa, and Pierluigi Vellucci. "Contrarian effect in opinion forming: insights from Greta Thunberg phenomenon." The Journal of Mathematical Sociology 47.2 (2023): 123-169.
However, misperception is a different "topic" from contrarians agents.

We thank the reviewer for suggesting the above literature . We now realize that the text was misleading, as we were not referring to ABM as methodology, but specifically to the works cited in the previous sentence. We changed the sentence and added the relevant literature. (line…). Furthermore, as pointed out by both reviewers, we also acknowledge that the use of the term “contrarians” was not accurately reflecting the characterization of our minoritarian agents. We therefore changed the definition also following the useful insight provided by the reviewer in the following comment (lines 83-87 in the Introduction, and then we uniformed the following sections).

2- Pag.2: Typo in the last sentence: "simulation works"

Corrected.

3- Pag.3: The first assumption of the model is b_i=+-1, constant in time. Does it mean that no one will change its opinion?

More specifically, what changes is the opinion that agents choose to express, while their belief (also referred as private opinion) stays constant in time. We provided a more extensive answer and justification above (please refer to the second point raised by the first reviewer).

4- Pag.5: The definition of contrarian agent does not fit with the definition in the literature. Maybe it is more appropriate to call them committed agents, or as the authors also suggested, "hard-cores". The main difference is that a contrarian agent has the opinion which is the opposite to a specific agent/group/majority, depending on which type of contrarian is considered, but it does not influence the strength of the belief.

We thank the reviewer for this useful suggestion. Indeed, we changed the term to incorporate the “hard-core” element which was missing. We now refer to such agents as

“hard-core minority”.

5- Pag.6: In order to improve the readability of the paper, the figures should be placed throught the text, above the caption. Moreover, the plots are blurred.

We improved the quality of the plots and figures. We placed the figures following the rules of the journal (the final position will change accordingly).

6- Pag.11: As a curiosity, do you think that the spiral of silence can be seen from the data?

To answer such a question, we tried to plot the percentage of silenced agents as a function of time for different values of K_c. The graphic shows a monotonic increase of the number of silenced agents up to its final value around 55%, which we already stated in the main text. Beside that, the plot is not very informative: differences among the different Kc are negligible, not only in the final value but also in the behavior over time (which is already visible in Figures 1(down) and 2(down): the dynamic is pretty fast – 15 time steps are enough to reach the final configuration). We therefore decided not to include the plot, as it would not add useful information to the manuscript.
